# Relationship between Pulmonary Function Tests and Severity of Coronary Artery Disease in Patients with Chronic Obstructive Pulmonary Disease

**Published:** 2020-11

**Authors:** Arsalan Salari, Fardin Mirbolouk, Asiyeh Ashouri, Pedram Salari, Hanie Shadrou, Zahra Mehdipour, Mahboobeh Gholipour

**Affiliations:** 1 Cardiovascular Diseases Research Center, Department of Cardiology, Heshmat Hospital, School of Medicine, Guilan University of Medical Sciences, Rasht, Iran,; 2 Research Center of Health and Environment, School of Health, Guilan University of Medical Sciences, Rasht, Iran

**Keywords:** Coronary artery disease, COPD, Pulmonary function test, SYNTAX score

## Abstract

**Background:**

The aim of this study was to examine relationship between the parameters of pulmonary function and the severity of coronary artery disease (CAD) in chronic obstructive lung disease patients.

**Materials and Methods:**

Four hundred and twenty four patients with ischemic heart diseases who underwent coronary angiography were studied. The demographic characteristics and medical history of the patients were obtained from their medical records.The severity of COPD was determined according to the Global Initiative for Chronic Obstructive Lung Disease. In addition, the severity of CAD was quantified by SYNTAX scoring.

**Results:**

Eighty-eight (21.2%), 270 (65.1%), 52 (12.5%), and 5 (1.2%) of the patients had the grade 1, 2, 3, or 4 COPD, respectively. In addition, 46 (11.1%), 319 (76.9%), and 50 (12.0%) of them had low, intermediate, and high CAD, respectively. A statistically significant relationship was observed between the severity of COPD and the severity of CAD. Significant relationships were found between age, sex, BMI, LDL, EF, and systolic pressure of pulmonary artery with the severity of COPD. The odds of higher CAD in females were 1.849 times higher than male patients. In addition, the odds of high CAD in the patients with grade 1 or 2 COPD were 0.006 and 0.068 times of the patients with grades 3 and 4 COPD, respectively.

**Conclusions:**

The findings of the present study indicate that the parameters of pulmonary function and the severity of CAD are associated with the severity of COPD.

## INTRODUCTION

Chronic obstructive pulmonary disease (COPD) is the presence of airflow obstruction which is not completely reversible. The airflow obstruction is progressive and may be accompanied by hyper-reactivity of airways to particles in the breathing air (1). The three types of COPD are emphysema, chronic bronchitis, and small airway (bronchioles) disease (1, 2). COPD is the fourth leading cause of mortality in the United States and the sixth in the world. It is estimated that among the most prevalent causes of mortality worldwide, COPD has raised to rank 3 (2, 3). The risk factors of COPD are respiratory infections, occupational exposure to hazardous materials such as the dusts and fumes of gold, silica, and coal, genetic factors such as α_1_-antitrypcin deficiency, air pollution, and smoking (4, 5). The symptoms and the functional limitations in COPD are because of the obstruction of airways, increased respiratory activity, and gas exchange disorders. The progression of the disease may lead to pulmonary hypertension, corpulmonale, and right-sided heart failure (5–7).

About 20% of COPD patients have no symptoms. However, the reduction of one-second Forced expiratory volume (FEV1), even at the normal range, may results in higher risks of cardiovascular incidents (8). Therefore, COPD is one of the risk factors of cardiovascular diseases (9–11). Previous studies showed that obesity, high levels of cholesterol, diabetes, and hypertension are important factors in the incidence of cardiovascular diseases (12). The prevalence of cardiac disorders in COPD patients is high, including pulmonary vascular disease, pulmonary hypertension, right-sided heart failure, arrhythmia, and Coronary artery disease (CAD) (13). For instance, Doucet et al. reported a high prevalence of ischemic heart diseases and heart failure in COPD patients (14). Likewise, Topsakal found significantly higher prevalence of CAD in 88 COPD patients in comparison with a control group (15). Similar findings have been reported by other authors (16, 17).

Cardiovascular diseases are the main cause of mortality worldwide, accounting for 50 and 25% of mortality in developed and developing countries, respectively (17). In addition, the prevalence of ischemic heart diseases is increasing with the CAD as the most common cardiovascular disease in the world (9, 18). Although the relationship between COPD and CAD is well-known, the responsible factors are not thoroughly identified (13). One of these factors is low-degree systemic inflammation in COPD patients that is related with increasing heart damages (13, 15). In fact, the levels of inflammatory factors such as C-reactive protein that play an important role in platelet accumulation and blood coagulation is increased in COPD patients because of heart failure (16). In addition, because of the reduction in the plasma oxidant capacity, low-degree chronic inflammation occurs which may be responsible for the high severity of atherosclerosis in COPD patients (15). Some studies reported the relationship between COPD and the progression of atherosclerosis (13–16).

Given the above, the present study was performed to assess relationship between the parameters of pulmonary function and the severity of CAD in COPD patients.

## MATERIALS AND METHODS

### Subject and study design

In this analytical cross-sectional study, 424 patients with ischemic heart diseases who underwent coronary angiography in the Heshmat Hospital, Rasht, north of Iran, were studied. The protocol of the study was approved by the Ethic committee of the Rasht University of Medical Sciences. Patients who were candidate for coronary angiography and bypass surgery, and those with the history of COPD and smoking were included in the study. Patients with respiratory diseases other than COPD, those with the history of previous angiographies, severe heart valve diseases, malignancies, and inflammatory diseases, as well as patients with liver or kidney insufficiency were excluded from the study.

### Data collection

Data were collected from the medical records of the patients. The obtained variables were age, sex, Body mass index (BMI), smoking, systolic and diastolic pressures, the levels of Low-density lipoprotein (LDL), FEV1, FEV1/FVC, Ejection fraction (EF), hemoglobin, hematocrit, white blood cells (WBC), and systolic pressure of pulmonary artery. In addition, echocardiography findings, including right ventricular size and function, Pulmonary artery systolic pressure (PASP), Tricuspid annular plane systolic excursion (TAPSE), as well as the history of taking β-2 agonist medications, anticholinergic factors, and inhalational steroids were recorded.

Patients with a FEV1/FVC ratio of less than 70% were considered as COPD patients and their grades of the disease was determined according to the Global Initiative for Chronic Obstructive Lung Disease (22) as follow: grade 1: FEV1≥0.8, grade 2: 0.5≤ FEV1<0.8, grade 3: 0.3≤ FEV1<0.5, and grade 4: FEV1<0.3. In addition, according to the angiography findings and SYNTAX scoring, the patients were assigned in three groups based on the levels of artery stenosis as follow: low artery stenosis: SYNTAX score ≤22, intermediate artery stenosis: 23 ≤SYNTAX score≤32, and high artery stenosis: SYNTAX score ≥33. In this scoring system, vessels with a diameter of 1.5 mm or more are examined and received scores only if 50% or more of the diameter is obstructed.

### Statistical analysis

Data were analyzed using version 21.0 of the SPSS software. Descriptive results are presented as frequencies (percent), mean ± standard deviation (SD), and median. The Kolmogorov-Smirnov goodness-of-fit test was used to assess the normality of the distribution of the variables. To assess the relationship between qualitative variables, the Chi-square test was used. The Spearman's correlation test was applied to assess the relationship between the severities of COPD and CAD with quantitative variables. The Kruskal-Wallis test was used to test the differences between the means of quantitative variables in the different levels of COPD or CAD. In addition, the relationship between the severities of COPD and CAD was assessed using the Kendall's tau correlation. To adjust potential confounding variables (age, sex, BMI, smoking, hemoglobin, systolic pressure of pulmonary artery), the multiple regression analysis was used. In all statistical tests, a p-value of less than 0.05 was considered significant.

## RESULTS

Of 424 patients, 9 were excluded from the study. Three hundred and ten (74.7%) patients were male and 105 (25.3%) were females. The majority of the patients (59.5%) were not smoker, 31% were smokers, and 9.5% were not current smokers, but had the past history of smoking. The average age and BMI of the patients were 60.11±9.11 years and 26.85±4.09 kg/m^2^, respectively. The medical characteristics of the studied patients including the levels of LDL, systolic and diastolic pressures, FEV1, FEV1/FVC, EF, hemoglobin, hematocrit, WBC, systolic pressure of pulmonary artery, TAPSE, and the history of diabetes are shown in table 1. About 44% of the patients had history of diabetes mellitus.

**Table 1.** Medical characteristics of the studied patients (n = 415)

**Variable**	**Min**	**Max**	**SD**	**Mean**	**Median**
**LDL (mg/dl)**	8.60	204.60	42.99	102.02	100.60
**SBP (mmHg)**	75.00	190.00	17.76	125.51	125.00
**DBP (mmHg)**	30.00	120.00	10.75	74.68	74.00
**FEV1**	0.94	4.67	0.66	2.39	2.40
**Predicted FEV1**	1.78	5.47	0.74	3.60	3.60
**FVC**	1.03	6.00	0.83	4.50	4.60
**FEV1/FVC (%)**	6.70	103.80	14.91	54.30	52.97
**EF (%)**	20.00	80.00	9.73	46.97	50.00
**Hb (g/dl)**	4.89	98.00	5.39	13.33	13.10
**Hct (%)**	19.50	93.50	6.78	40.15	39.70
**WBC (per μl)**	700	40400	3324	8839	8200
**Systolic pressure of pulmonary artery (mmHg)**	16.00	46.00	5.08	29.01	29.00
**TAPSE (cm)**	1.40	28.00	2.89	2.66	2.10
**Diabetes**	N		%		
	179		43.7		

The assessment of the patients by the GOLD indicated that 88 (21.2%), 270 (65.1%), 52 (12.5%), and 5 (1.2%) of them had the grades 1, 2, 3, or 4 COPD, respectively. In addition, the assessment of the severity of CAD by SYNTAX scoring showed that 46 (11.1%), 319 (76.9%), and 50 (12.0%) of the patients had the low, intermediate, and high artery stenosis, respectively.

Table 2 shows the relationship between the severities of COPD and CAD in the studied patients. Among patients with grade 1 COPD only 1 (1.1%) had high CAD. On the other hand, all patients with grade 4 of COPD had the high level of CAD. A statistically significant relationship was observed between the severity of COPD and the severity of CAD. The Kendall's tau correlation showed a statistically significant linear correlation between the severities of COPD and CAD so that patients with higher grades of COPD had higher levels of CAD.

**Table 2.** Relationship between the severities of COPD and CAD in the studied patients (n = 415)

Variable	Grade	CAD severity				Chi-square statistics^*^	p-value

		Weak	Intermediate	High	Total		
COPD severity	1	34 (38.6%)	53 (60.2%)	1 (1.1%)	88	195.77 (df=6)	<0.001
	2	12 (4.4%)	239 (88.5%)	19 (7%)	270		
	3	0	27 (51.9%)	25 (48.1%)	52		
	4	0	0	5 (100%)	5		
Kendall's tau correlation coefficient		0.520					<0.001

* Obtained from Chi-square-Pearson statistics

Table 3 indicates the relationship between demographic and medical characteristics of the patients with the severity of COPD. The Kruskal-Wallis test showed significant relationships between age, sex, BMI, LDL, EF, and systolic pressure of pulmonary artery with the severity of COPD. Female patients had higher severity of COPD than males. Similarly, older patients had higher grades of the disease. The mean of EF in the patients with grade 2 was significantly higher than those with grades 3 and 4 COPD (p=0.12). In contrast, no statistically significant differences were noted in the means of EF between the patients with grade 1 or 2 and between the patients with grade 1 and those with grade 3 and 4 COPD. No significant relationships were found between systolic and diastolic pressures, smoking, hemoglobin, hematocrit, WBC, TAPSE, and the history of diabetes with the severity of COPD. The Spearman's correlation test showed significant association between the severity of COPD and age, BMI, LDL, EF, and systolic pressure of pulmonary artery. However, the relationship was weak with correlation coefficients between 0.1 and 0.3.

**Table 3.** Spearman correlation between demographic and medical characteristics with the severities of COPD and CAD

**Variables**		**COPD severity**			**p-value**	**COPD severity**		**CAD severity**	
	
		Grade 1	Grade 2	Grade 3 or 4		Correlation	p-value	Correlation	p-value
**Age (yr)**		58.31±9.29	60.01±8.89	63.35±9.15	.005	.152^**^	.002	.130**	.008
**BMI (kg.m^2^)**		26.11±3.8	26.91±4.23	27.75±3.7	.035	.124^*^	.012	.112*	.023
**LDL (mg/dl)**		92.31±38.37	102.49±42.37	114.71±49.33	.012	.147^**^	.004	−.053	.295
**SBP (mmHg)**		125.89±16.93	124.95±18.5	127.61±15.29	.395	.029	.564	.084	.089
**DBP (mmHg)**		75.36±10.05	74.66±10.86	73.67±11.38	.481	−.051	.304	−.032	.523
**FEV1**		2.92±0.62	2.38±0.55	1.63±0.38	<.001	−.550^**^	<.001	−.389^**^	<.001
**Predicted FEV1**		3.39±0.73	3.64±0.74	3.74±0.72	.003	.159^**^	.001	.021	.669
**FVC**		4.12±0.93	4.57±0.78	4.72±0.72	<.001	.220^**^	<.001	−.001	.988
**FEV1/FVC (%)**		69.79±12.52	52.96±10.95	36.76±11.19	<.001	−.684^**^	<.001	−.407**	<.001
**EF (%)**		46.08±10.07	47.7±9.68	44.86±9.12	.029	−.025	.615	−.018	.709
**Hb (g/dl)**		13.38±1.57	13.43±6.57	12.82±1.79	.112	−.097^*^	.047	−.198^**^	<.001
**Hct (%)**		40.58±4.41	40±7.01	40.19±8.63	.295	−.072	.146	−.142^**^	.004
**WBC (per μl)**		8788 ±2598	8804 ±3161	9084 ±4806	.855	−.025	.610	.030	.546
**Systolic pressure of pulmonary artery (mmHg)**		28.36±4.44	28.76±5.01	31.23±5.79	.006	.137^**^	.005	.315^**^	<.001
**TAPSE (cm)**		2.17±0.37	2.12±0.35	2.17±0.38	.494	−.015	.769	−.097	.054
**Sex**	Male	81(26.1)	191(61.6)	38(12.3)	<0.001				
	Female	7(6.7)	79(75.2)	19(18.1)					
**Smoking**	No	43(17.8)	159(65.7)	40(16.5)	0.1				
	Yes	34(27)	81(64.3)	11(8.7)					
**Diabetes**	No	52(22.5)	152(65.8)	27(11.7)	0.39				
	Yes	36(20.1)	114(63.7)	29(16.2)					

* Obtained from Kruskal-Wallis test;

** Obtained from Chi-square test.

Table 4 shows the relationship between the severities of COPD and CAD in the studied patients. After controlling the confounding variables (sex, age, BMI, smoking, hemoglobin, and systolic pressure of the pulmonary artery), the regression model indicated a significant relationship between the severities of COPD and CAD (p<0.01). According to the goodness-of-fit and the Cox-Snell R2, two models were found as the best predictors of the severity of CAD in the COPD patients. Model 1 includes the severity of COPD and the sex of the patients. Model 2 includes the severity of COPD and systolic pressure of pulmonary artery. According to the model 1, the odds of higher CAD in females were 1.849 times higher than male patients. In addition, the odds of higher CAD in the patients with grades 1 or 2 COPD were 0.006 and 0.068 times of the patients with grades 3 and 4 COPD, respectively (Cox-Snell R^2^= 0.30). According to the model 2, one unit increase in the systolic pressure of the pulmonary artery increases the severity of CAD by 1.18 times. In addition, the odds of higher CAD in the patients with grades 1 or 2 COPD were 0.006 and 0.074 times of the patients with grades 3 and 4 COPD, respectively (Cox-Snell R^2^= 0.35).

**Table 4.** Logistic regression analysis of the relationship between the severities of COPD and CAD in the studied patients (n=415)

Variable		Odd ratio	95% CI	p-value	Goodness-of-fit		Cox-Snell R2

					Chi-square statistic (df)	p-value	
Model 1	SPPA	1.183	1.118–1.251	<0.001	81.86 (115)	0.91	0.35
	Grade 1 COPD	0.005	0.002–0.014	<0.001			
	Grade 2 COPD	0.074	0.038–0.155	<0.001			
Model 2	Sex (female)	1.849	3.422–1.0	0.05	2.66 (df=7)	0.99	0.30
	Grade 1 COPD	0.006	0.002–0.015	<0.001			
	Grade 2 COPD	0.068	0.034–0.137	<0.001			

## DISCUSSION

In the present study we investigated the relationship between the parameters of pulmonary function and the severity of CAD in COPD patients. Coronary artery disease and COPD are common diseases in the adult population. Our study of 415 COPD patients showed that there were statistically significant relationships between sex, age, BMI, LDL, EF, and systolic pressure of pulmonary artery with the severity of COPD. In the present study, female patients had higher grades of COPD than males. In contrast, previous studies reported higher severity of COPD in men than women (19–21). This controversy may be because of the higher sensitivity of female patients in the present study to irritant and toxic agents that resulted in a severe shortness of breath at the initiation of activity. This is why female COPD patients have a lower quality of life than males (22).

A number of studies showed that smoking is one of the risk factors that increase the odds of COPD (23, 24). However, we did not find a significant relationship between smoking and the severity of COPD. This contrast may be due to the cross sectional nature of our study, and in order to assay the influence of smoking, longitudinal studies is required.

COPD patients can be classified according to the GOLD guideline into four groups based on the reduction of FEV1 in relation to the predicted value of it. Many studies have shown COPD as a risk factor for cardiovascular diseases and have found that COPD is associated with cardiovascular mortality; these studies indicated that the more severe the COPD, the greater the likelihood of CAD in these patients (25, 26). In addition, cardiovascular diseases are important predictors for hospitalization and mortality of COPD patients (25). In the present study, a significant relationship between the severities of COPD and CAD was observed. In our study, the significant relationship between the severity of COPD and the level of CAD remained even after controlling the effects of confounding variables. In parallel, a study by Dursunoglu et al. demonstrated that multivessel disease is more in patients with COPD (16), in another study by Zhu et al. it was also shown that COPD was associated with severity of CAD (27). Other study also reported that the more severe the COPD, the more the mortality after percutaneous coronary intervention (17). The reason for this relationship in our study may be due to the increased level of carbon dioxide in COPD patients which can play an important role in the progression of atherosclerosis and then increasing the probability of coronary artery disease.

We found statistically significant relationship between the severity of CAD and sex, age, smoking, FEV1, FEV1/FVC, hemoglobin, hematocrit, and the systolic pressure of pulmonary artery. In the present study, female patients had higher severity of CAD, a finding that is in line with that of Hochner-Celnikier et al (23). In contrast, some studies showed higher prevalence of CAD and its severity in male COPD patients (23, 28, 29). Age is one of the risk factors of cardiovascular diseases, including CAD. In the present study, younger patients had lower severity of CAD than older patients. In contrast, some studies reported that the severity of CAD increases by age (30). It seems that the direct relationship between age and CAD is because of the atherosclerosis which occurs and progresses with age. In addition, in contrast to most of previous studies (21, 31–34), smoker patients had lower severity of CAD.

In the present study, history of diabetes and higher levels of systolic pressure of pulmonary artery was proportional to higher severity of CAD. Some studies reported hypertension and diabetes as the main causes of CAD (34–37). For instance, Blomström-Lundqvist et al., found that 58% of the CAD patients had hypertension (38). In addition, Curkendall et al. reported that the prevalence of CAD among hypertensive patients was 74% (39). These results are in consistent with our findings. In fact, pulmonary hypertension is associated with decreased survival in COPD patients. Hypoxia seen in pulmonary hypertension patients plays a role both in COPD and CAD.

## CONCLUSION

The findings of the present study indicate that the parameters of pulmonary function and the severity of CAD are associated with the severity of COPD and they can be considered as predictors in patients with different severities of COPD.

### Limitations of the Study

Our study suffers from some limitations. First of all it was a cross sectional study and in this type of study we could not prove a cause-consequence relationship. Another limitation may be the small sample size of the study which may have affected the results; in order to obtain better results we suggest establishment of larger prospective studies.
